# 

**Published:** 1869-06

**Authors:** 


VOL. XXIII. No. 6.

### THE

### Dental Register.

EDITED BY

J. TAFT & G. WATT.

JUNE, 1869.

MONTHLY:

THREE DOLLARS PER ANNUM, IN ADVANCE.

CINCINNATI:
WRIGHTSON & CO., Printers, 167 WALNUT STREET.

J. & WM. TAFT, Dentists, 117 West Fourth St.

